# Non-Operative Management of Esophageal Cancer with Complete Clinical Response After Neoadjuvant Therapy: Current Status and Future Directions

**DOI:** 10.3390/jpm16060317

**Published:** 2026-06-13

**Authors:** Sofia Bertona, Javier Castillo, Francisco Schlottmann

**Affiliations:** 1 Department of Surgery, Hospital Alemán of Buenos Aires, Buenos Aires C1118AAT, Argentina; 2 Department of Medical Oncology, Hospital Alemán of Buenos Aires, Buenos Aires C1118AAT, Argentina; 3 Department of Surgery, University of Illinois at Chicago, Chicago, IL 60612, USA

**Keywords:** esophageal cancer, neoadjuvant therapy, non-operative management, organ preservation, clinical complete response, pathological complete response, active surveillance

## Abstract

**Introduction**: Esophagectomy has traditionally been considered mandatory after neoadjuvant therapy for locally advanced esophageal cancer. However, recent evidence has challenged this paradigm and motivated interest in organ-preservation strategies with active surveillance in patients achieving clinical complete response (cCR). **Methods**: A literature search was performed using PubMed/MEDLINE, ScienceDirect, and Embase databases to identify relevant studies related to non-operative management (NOM) of esophageal cancer. Evidence was synthesized qualitatively with a critical focus on the biological rationale of NOM, diagnostic limitations of response-assessment, oncologic outcomes associated with surveillance strategies and the evolving role of molecular biomarkers. **Results**: The safety of NOM with active surveillance is tightly linked to the diagnostic accuracy of response assessment. Although structured multimodal response assessment protocols combining endoscopy, endoscopic ultrasound, and PET-CT have shown acceptable performance, residual clinically undetectable disease might persist in some patients. Evidence from the SANO trial has suggested non-inferior short-term survival outcomes of NOM compared with immediate esophagectomy in carefully selected patients with cCR after neoadjuvant chemoradiotherapy treated within specialized centers. Nevertheless, long-term oncologic outcomes remain unknown, and uncertainty persists regarding the broader applicability of this strategy outside specialized multidisciplinary settings. Emerging biomarker-driven approaches including PD-L1 expression, microsatellite instability, and circulating tumor DNA (ctDNA) may further refine response assessment and help identify patients most suitable for organ-preservation strategies. **Conclusions**: Active surveillance represents a promising alternative to immediate esophagectomy in selected patients with cCR after neoadjuvant therapy. However, further studies with longer follow-up and standardized surveillance protocols are still needed to safely implement this strategy outside trial settings.

## 1. Introduction

The prognosis of esophageal cancer remains unfavorable in most patients due to its fast dissemination and advanced stage when diagnosed, with overall 5-year survival of 27–39%. However, overall survival of patients with locally advanced esophageal cancer has still improved after the introduction of neoadjuvant therapies [1,2,3].

For patients with locally advanced resectable disease, neoadjuvant chemoradiotherapy (nCRT) followed by esophagectomy has become one of the standard therapies, largely driven by the survival benefit demonstrated in the CROSS trial. This study demonstrated that nCRT significantly improved overall survival compared with surgery alone and resulted in a pathologic complete response (pCR) in approximately 29% of patients, with even higher rates observed in squamous cell carcinoma (49% squamous cell carcinoma vs. 23% adenocarcinoma) [4,5]. While pCR refers to the absence of viable tumor cells in the surgical specimen, clinical complete response (cCR) is defined by the absence of detectable residual disease on post-treatment clinical assessment.

The recognition that up to one-third of patients may achieve pCR after nCRT has prompted growing interest in organ-preservation strategies and raised questions regarding the necessity of immediate surgery in selected patients. Esophagectomy is indeed associated with relatively high perioperative morbidity and mortality, along with a potential impact on quality of life [6,7,8]. Consequently, the question has emerged whether immediate surgery is necessary in all patients who achieve complete clinical response (cCR) after nCRT. Therefore, an active surveillance strategy has been proposed for patients with cCR [9].

However, major uncertainties remain regarding which patients can safely avoid esophagectomy and whether surveillance can maintain long-term oncologic safety. The appeal of active surveillance therefore depends on the probability and the patterns of recurrence because salvage surgery is potentially curative only when failure remains locoregional and technically resectable. The concept of organ preservation in esophageal cancer did not arise from a single transformative trial but rather evolved progressively from observations embedded within trimodality therapy studies.

In this narrative review, we critically examine the biological rationale, diagnostic challenges, oncologic outcomes, and current controversies surrounding active surveillance after nCRT for esophageal cancer. We aimed to contextualize recent randomized evidence within surgical practice and to discuss whether organ preservation may represent a feasible strategy for carefully selected esophageal cancer patients within structured surveillance programs.

## 2. Methods

This study was conducted as a scoping narrative review with a literature search performed in PubMed/MEDLINE, ScienceDirect, and Embase databases. The search included a combination of keywords and Medical Subject Headings (MeSH) such as esophageal cancer, watch and wait, active surveillance, non-operative management, clinical complete response, neoadjuvant chemoradiotherapy, preoperative chemotherapy, organ preservation, immunotherapy, microsatellite instability, PDL-L1 and circulating tumor DNA. Additional articles were identified through manual screening of reference lists from selected publications.

The literature search included studies published between January 2003 and March 2026. Two authors (SB and FS) independently performed literature screening and study selection. Priority was given to high-level evidence including randomized clinical trials and retrospective or prospective cohort studies. Case reports, conference abstracts, editorials, and studies with insufficient outcome data were excluded. Systematic reviews and meta-analyses were screened for additional relevant references. Only studies published in English were considered.

The initial search identified approximately 271 articles. After title and abstract screening and full-text review, 52 studies were considered relevant and included in the qualitative synthesis. The studies were then prioritized according to methodological quality, sample size, clinical relevance, and applicability to contemporary multimodal treatment strategies in esophageal cancer. Given the narrative nature of the review, no formal quality assessment or quantitative meta-analysis was performed.

Emphasis was placed on prospective response-assessment trials (pre-SANO), randomized surveillance studies (SANO) and perioperative immunotherapy trials with potential implications for organ-preservation pathways. Evidence was synthesized qualitatively with a critical focus on the biological rationale of NOM, diagnostic limitations of response assessment, oncologic outcomes associated with surveillance strategies and the evolving role of molecular biomarkers such as PD-L1 expression, microsatellite instability and circulating tumor DNA.

## 3. Current Evidence

### 3.1. Biological Rationale for Organ Preservation

Historically, esophagectomy was considered mandatory following nCRT, irrespective of tumor response in locally advanced disease. This paradigm was built upon the assumption that residual microscopic disease might inevitably persist after nCRT and that surgical resection was required to achieve durable oncologic control. This paradigm was challenged by the substantial pCR rates observed after modern multimodal therapies.

The organ-preservation strategy was initially developed in rectal cancer. With the adoption of total neoadjuvant therapy (TNT), NOM with a watch-and-wait strategy has emerged as a feasible approach in carefully selected patients achieving cCR [10]. Several studies have demonstrated that deferral of radical surgery (i.e., total mesorectal excision) in these patients does not appear to compromise overall survival or distant oncologic outcomes compared with immediate resection [11,12].

In addition to avoiding radical surgery, organ-preserving approaches have been associated with improved functional outcomes and better patient-reported quality of life [13]. These observations have stimulated growing interest in extending organ-preservation strategies to other organs such as the esophagus, particularly given the significant morbidity and mortality associated with esophagectomy. Consequently, the possibility that a subset of patients achieving cCR after neoadjuvant therapy might safely avoid surgery has prompted the investigation of NOM strategies in esophageal cancer. Nevertheless, caution is warranted when extrapolating data from rectal cancer, as important differences in tumor biology, treatment response, and patterns of recurrence may limit the direct applicability of these concepts to esophageal malignancies.

The CROSS trial established nCRT as one of the standard neoadjuvant therapies and demonstrated pCR rates of approximately 29% (reaching 49% in squamous cell carcinoma). Long-term follow-up confirmed durable survival benefits with multimodal therapy, but also revealed that a significant subset of patients had no residual tumor at resection, raising concern that mandatory surgery might overtreat selected responders [4,5,14,15].

Accurate identification of cCR became critical for NOM strategies. The Dutch pre-SANO trial was a prospective, multicenter diagnostic cohort study designed to evaluate the accuracy of cCR assessment following nCRT. The trial used a structured multimodal protocol including endoscopy with bite-on-bite biopsies, EUS-guided fine-needle aspiration of suspicious lymph nodes, and positron emission tomography–computed tomography (PET-CT) imaging for interval metastases. The study demonstrated that approximately 90% of clinically relevant residual tumors could be detected with those diagnostic methods (thereby small foci of residual microscopic disease could still remain undetected). Importantly, pre-SANO was not designed to evaluate surveillance as a therapeutic strategy, but rather to quantify the diagnostic performance of response-assessment tools. These findings provided the methodological foundation for subsequent surveillance trials [16,17].

The randomized SANO trial represents the first non-inferiority study comparing active surveillance with immediate surgery in patients showing cCR after nCRT. At a median follow-up of approximately two years, overall survival in the surveillance group was non-inferior to that observed in the standard surgery group within the prespecified non-inferiority margin. Notably, nearly half of patients assigned to surveillance avoided esophagectomy during the study period, while most cases of locoregional regrowth were promptly detected and managed with salvage surgery [18].

Interpretation of these findings, however, requires extreme caution. The follow-up duration remains relatively short, and the study was conducted within highly specialized centers employing strict response-assessment protocols. Furthermore, non-inferiority in short-to-midterm survival does not exclude potential differences in long-term outcomes or distant recurrence patterns. Therefore, while SANO provides the strongest evidence to date supporting structured surveillance in carefully selected responders, its generalizability outside controlled multidisciplinary settings warrants careful consideration. Successful implementation of NOM therefore depends on careful patient selection, intensive surveillance protocols, access to advanced diagnostic modalities, and availability of experienced salvage surgical team.

Trials adding immunotherapy to a chemotherapy backbone, such as KEYNOTE-585, DANTE, and MATTERHORN, demonstrated improved pathologic response rates in gastroesophageal malignancies. Molecularly selected cohorts demonstrated substantially higher response rates, with microsatellite instability-high (MSI-H) tumors achieving 58–60% pCR after dual immune checkpoint blockade [19,20,21,22]. Simultaneously, the ESOPEC trial demonstrated improved survival with perioperative chemotherapy with FLOT compared with nCRT in resectable esophageal adenocarcinoma. While not designed to evaluate surveillance, these results challenge the universality of organ-preservation protocols developed in the context of chemoradiotherapy and suggest that the biological rationale for NOM may differ according to the selected systemic treatment backbone, further supporting the need for individualized treatment strategies rather than broad application of organ-preservation approaches [23].

The pivotal studies that have shaped the evolution of organ-preservation strategies are chronologically summarized in Table 1.

### 3.2. Clinical Complete Response: Definition and Limitations

Clinical complete response (cCR) refers to the absence of detectable residual disease on post neoadjuvant therapy clinical evaluation (e.g., endoscopy, EUS, PET-CT). Pathologic complete response (pCR) is defined by the absence of viable tumor cells in the resection specimen (i.e., ypT0N0), which correlates with a superior disease-free survival and overall survival [27]. The safety and feasibility of organ preservation ultimately rests on how closely cCR mirrors true pCR.

The pre-SANO trial [16] demonstrated that a structured protocol combining endoscopy with bite-on-bite biopsies, EUS-guided nodal sampling, and PET-CT imaging can detect the majority of clinically relevant residual tumors. However, even with the use of such a strict protocol, a proportion of residual disease remains undetected. For instance, approximately 10% of clinically relevant residual tumors were missed despite multimodal assessment. Furthermore, EUS and PET-CT demonstrated false-negative rates of 28% and 15% for locoregional residual disease, respectively. Treatment-induced fibrosis, dispersed tumor regression (particularly in adenocarcinoma), and unavoidable sampling error reduce the accuracy of endoscopic assessment. Exclusion of residual nodal disease also remains challenging with current imaging techniques, as some patients in pre-SANO demonstrated positive nodal fine-needle aspiration despite negative endoscopic biopsies [16].

In parallel, the INFINITY trial evaluated a biomarker-driven NOM strategy in patients with MSI-high gastric and gastroesophageal junction adenocarcinoma treated with neoadjuvant immunotherapy. In this study, response reassessment was performed between weeks 12 and 14 using a structured multimodal approach that included the same diagnostic methods as the pre-SANO trial, adding a contrast-enhanced chest–abdomen–pelvis computed tomography and circulating tumor DNA (ctDNA). The latter was integrated into the surveillance protocol every 12 weeks for up to two years [22].

While substantial progress has been made in refining response evaluation with the use of liquid biopsy, the identification of true cCR remains based on probability rather than definitive evidence of tumor eradication. Active surveillance should therefore only be conceptualized within a structured, risk-adapted monitoring strategy designed to detect regrowth at a curable stage.

**Figure 1:**
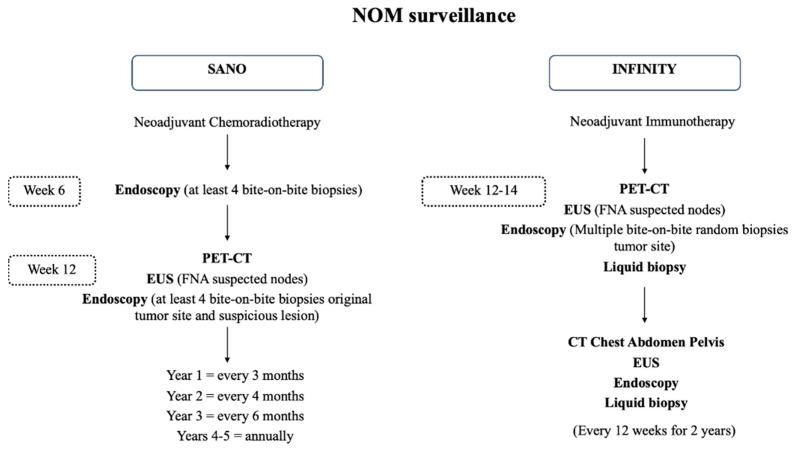
Potential follow-up protocols for non-operative management in patients with esophageal cancer. (Abbreviations: NOM, non-operative management; EUS, endoscopic ultrasound; FNA, fine-needle aspiration; PET-CT, positron emission tomography–computed tomography; CT, computed tomography).

Figure 1 describes potential surveillance protocols for patients with esophageal cancer undergoing non-operative management.

### 3.3. Oncological Outcomes

The oncologic safety of active surveillance in esophageal cancer has been progressively evaluated across different studies. The SANO trial provides the highest level of evidence to date. At a median follow-up of two years, overall survival in the surveillance group was non-inferior to that observed in the standard surgery group within the prespecified non-inferiority margin, although equivalence between strategies cannot yet be assumed [18]. Notably, nearly half of patients assigned to surveillance avoided esophagectomy during the study period. As previously discussed, the non-inferiority design, limited follow-up with no mature long-term overall survival or distant recurrence data currently available, and implementation within strictly protocolized high-volume centers necessitate careful consideration when extrapolating these results to broader clinical practice.

Other observational studies have reported similar results. A Dutch multicenter propensity matched analysis reported no significant difference in overall survival between active surveillance and immediate surgery among patients with cCR after neoadjuvant therapy [28]. Similarly, retrospective cohort analyses have failed to demonstrate statistically significant survival disadvantages associated with surveillance [29,30]. Although observational studies have reported comparable survival outcomes, these findings may be influenced by substantial selection bias, immortal time bias and other inherent methodological limitations of non-randomized designs.

Locoregional regrowth represents the principal oncologic concern after an organ-preservation strategy. Prospective surveillance cohorts have consistently demonstrated that most regrowth events occur within the first two years of follow-up and are predominantly locoregional [28,31,32]. Importantly, the majority of these patients underwent successful salvage esophagectomy, with acceptable perioperative morbidity and encouraging oncologic outcomes in experienced high-volume centers [31,32]. In the SANO trial, postoperative morbidity and 90-day mortality rates after postponed surgery were similar to those observed after standard surgery (including anastomotic and pulmonary complications) [18]. Nevertheless, delayed resection after chemoradiotherapy remains technically demanding, mostly due to extensive radiation-induced fibrosis and adhesions, which highlights the importance of surgical expertise. Overall, these findings support the role of early detection protocols to enable timely and safe salvage surgery in highly experienced centers without compromising short-term overall survival [18].

Distant metastases, on the other hand, remain the predominant pattern of failure in esophageal cancer, irrespective of the timing of surgery. Available comparative data have not demonstrated a clear increase in distant recurrence among patients managed with structured surveillance [18,28,30]. Nonetheless, longer follow-up is required to determine whether delayed surgical intervention influences systemic relapse patterns or late oncologic outcomes.

Concerns regarding delayed resection after neoadjuvant therapy have also been raised in previous research evaluating surgical timing. Several studies have reported that prolonged intervals between neoadjuvant therapy and esophagectomy may be associated with increased perioperative morbidity and mortality and, in some cohorts, inferior overall survival [33,34,35]. The JCOG1109 trial further suggested that extended intervals between neoadjuvant therapy and surgery may adversely affect oncologic outcomes in esophageal squamous cell carcinoma. These findings underscore the importance of strict response-assessment protocols, close monitoring, and timely salvage intervention when NOM strategies are pursued [36].

Overall, oncologic and surgical safety of active surveillance remains under debate and definitive long-term equivalence to immediate surgery has not yet been established. Its safety likely depends on meticulous patient selection and delivery within specialized centers with timely access to salvage surgery.

### 3.4. Patient Selection: Who Is the Ideal Candidate?

Active surveillance should not be considered as an alternative to surgery, but rather as a conditional strategy whose safety and effectiveness rely on three main pillars:

- Tumor-related factors (tumor biology and response characteristics);
- Institutional diagnostic and surveillance capabilities;
- Patient-specific risks and preferences.

Active surveillance should be considered only in patients with a rigorously confirmed cCR after neoadjuvant therapy, assessed within structured and standardized response-evaluation protocols. The safety of this approach is thereby tightly linked to the diagnostic accuracy of response assessment (e.g., pre-SANO multimodal protocol to reduce false-negative results) [16]. Therefore, patients evaluated outside structured, protocol-driven environments may not benefit from the same oncologic safety.

The histologic subtype might also influence the safety of this approach. In the CROSS trial, SCC demonstrated substantially higher pCR rates following neoadjuvant chemoradiotherapy compared with adenocarcinoma (49% vs. 23%) [5]. For this reason, SCC might be associated with favorable oncologic outcomes among carefully selected clinical complete responders [37]. By contrast, esophageal adenocarcinoma often demonstrates heterogeneous tumor regression patterns after neoadjuvant chemoradiotherapy, which have been associated with a higher risk of recurrence and worse prognosis (fragmented patterns may reflect dispersed residual tumor elements). This morphological heterogeneity reduces the sensitivity of endoscopic biopsies for detecting residual disease and thereby increases the risk of false-negative results [16,38]. These histology-specific differences are also critical when extrapolating surveillance strategies across tumor types. Accordingly, future response-adapted surveillance protocols and selection criteria might require histology-specific refinements. Unfortunately, the available evidence is still insufficient to establish different surveillance protocols according to histology.

Esophagectomy remains associated with high perioperative morbidity even in high-volume centers, particularly in elderly or frail/comorbid patients [39]. In such individuals, the relative benefit of organ preservation is amplified, provided that surveillance can be delivered within experienced multidisciplinary programs. Patient preference analyses have also demonstrated substantial heterogeneity regarding patient considerations, with a proportion of individuals willing to accept an increased risk of locoregional recurrence in order to avoid immediate esophagectomy and its associated potential morbidity [40].

**Figure 2:**
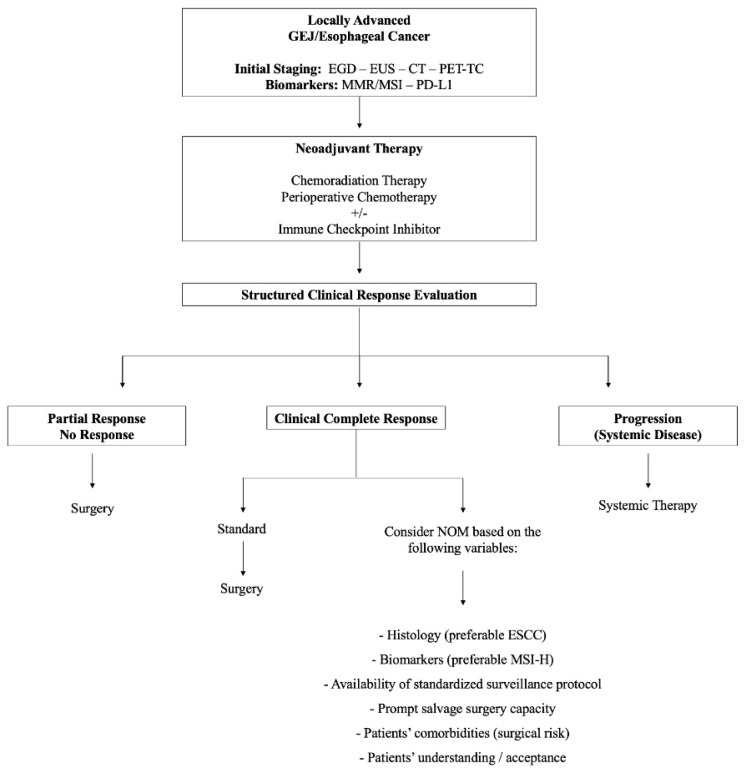
Algorithm for selecting potential candidates for an organ-preservation strategy in esophageal cancer. (Abbreviations: GEJ, gastroesophageal junction; EGD, esophagogastroduodenoscopy; EUS, endoscopic ultrasound; CT, computed tomography; PET-CT, positron emission tomography–computed tomography; MMR/MSI, mismatch repair/microsatellite instability; PD-L1, programmed death-ligand 1; NOM, non-operative management; ESCC, esophageal squamous cell carcinoma; MSI-H, microsatellite instability-high).

Overall, we believe that a structured, shared decision-making process within multidisciplinary settings remains critical during patient selection for active surveillance strategies (Figure 2).

### 3.5. Emerging Biomarkers and Response-Adapted Strategies

Future progress in organ-preservation strategies for esophageal cancer will likely depend on improving the precision of response assessment and refining patient selection through the integration of emerging imaging technologies, molecular biomarkers, and immune-related predictors of treatment response.

Radiomics-based approaches and artificial intelligence-driven image analysis have shown promising potential in predicting treatment response and identifying imaging patterns associated with residual disease. Early studies suggest that quantitative features extracted from CT imaging may improve the prediction of pCR [41,42].

The use of liquid biopsy is also emerging as a potential complement to conventional surveillance. Circulating tumor DNA (ctDNA) has demonstrated the ability to detect minimal residual disease and may identify patients at a higher risk of recurrence despite an apparent clinical (endoscopy, EUS, CT, and/or PET-CT) complete response [43,44]. Several studies have shown that detectable ctDNA after systemic therapies is strongly associated with disease progression, suggesting that molecular monitoring could improve early detection of occult disease and inform risk-adapted surveillance strategies [45,46].

Tumor-specific biological characteristics may also influence treatment response and the feasibility of organ preservation. Immune-related biomarkers, including programmed death-ligand 1 (PD-L1) expression and microsatellite instability (MSI) status, have emerged as potential predictors of response to immune checkpoint inhibition [47,48]. While MSI-high tumors are relatively uncommon in esophageal cancer, their presence identifies a biologically distinct subgroup characterized by increased mutational burden and enhanced immune activation, which may confer a heightened sensitivity to immunotherapy [48]. In the NEONIPIGA study, neoadjuvant nivolumab combined with ipilimumab achieved pCR rates approaching 60%, with excellent short-term survival outcomes. In addition, a small subset of patients achieving a clinical complete response who declined surgery remained disease-free during follow-up [26]. Similarly, the INFINITY trial extended these findings, prospectively testing NOM, reporting pCR rates of 60% (80% of patients with major pathologic response) and favorable progression-free survival and OS (cCR rates of 76% in the surveillance cohort) [22]. Together, these studies suggest that, in carefully selected subgroups, cCR in MSI-H disease after immunotherapy-based treatment may more closely approximate pCR. However, longer follow-up and larger prospective studies are required before these approaches can be safely incorporated into routine clinical practice.

Similarly, tumors with higher PD-L1 expression have demonstrated survival benefits [49]. The incorporation of immune checkpoint inhibitors into perioperative treatment strategies has further strengthened the biological rationale for organ-preservation approaches in esophageal cancer. By enhancing tumor regression and improving systemic disease control at an early stage of treatment, immunotherapy-based regimens might increase the proportion of patients achieving complete clinical responses potentially eligible for NOM. Early-phase evidence from the PALACE-1 trial demonstrated pCR rates of up to 55.6% with the addition of pembrolizumab to neoadjuvant chemoradiotherapy in esophageal SCC, supporting the hypothesis that intensified multimodal therapy may expand the pool of candidates for surveillance-based strategies [25]. Complementary evidence from the adjuvant setting, including the CheckMate 577 trial, has demonstrated improved disease-free survival with nivolumab after neoadjuvant chemoradiotherapy and surgery, highlighting the importance of systemic immune control in reducing the recurrence risk [24]. Similarly, trials such as the KEYNOTE-585 (improved pCR), DANTE (improved pCR), and MATTERHORN (improved pCR and 2-year event-free survival) further explored the role of immunotherapy in improving response depth and long-term oncologic outcomes in upper gastrointestinal malignancies [19,20,21]. Collectively, these developments support a shift toward response-adapted treatment strategies in which improved systemic control and deeper tumor regression may facilitate organ preservation while maintaining oncologic safety in carefully selected patients.

The SANO-3 study (SANO-3) is a non-randomized phase II trial evaluating the efficacy of maintenance nivolumab during active surveillance in patients with cCR after neoadjuvant chemoradiation, with the aim of reducing the incidence of both locoregional regrowth and distant relapse [50]. This approach represents a conceptual extension of active surveillance into the immunotherapy era by targeting minimal residual disease at a molecular level.

The future of active surveillance will likely depend on a more integrated, biomarker-driven approach combining advanced imaging, molecular monitoring, and immune profiling. Prospective studies incorporating these tools will be essential to determine whether such strategies can safely expand the population of patients eligible for organ preservation without compromising long-term oncologic outcomes.

Overall, biomarker-driven NOM strategies are promising but remain investigational and should therefore still be tested within trial settings.

### 3.6. Challenges of Active Surveillance in Esophageal Cancer

Despite the growing interest in active surveillance strategies, several important limitations and challenges must be considered when implementing a “watch-and-wait” approach in esophageal cancer.

One of the main concerns relates to the limited accuracy of current available methods to assess clinical response. Endoscopy, EUS, and metabolic imaging (i.e., PET-CT) remain imperfect tools for detecting microscopic residual disease, and false-negative assessments may lead to delayed surgical treatment in patients with disease persistence. Additionally, the success of surveillance strategies relies on strict follow-up protocols, availability of multidisciplinary teams capable of performing repeated evaluations and timely salvage surgery when required, and sustained patient adherence to intensive surveillance. Variability in institutional infrastructure, imaging interpretation and endoscopic expertise may further limit the reproducibility of surveillance outcomes outside specialized high-volume centers. The addition of liquid biopsies with ctDNA will likely improve the sensitivity of response assessment, but further studies with larger numbers of patients and longer follow-up are needed.

Current surveillance strategies are largely based on the intensive protocols established in the pre-SANO, SANO, and INFINITY studies, particularly during the first two years after treatment when the risk of locoregional regrowth is highest. In the INFINITY trial, surveillance was further complemented with serial ctDNA monitoring every 12 weeks for up to two years. However, the optimal duration and intensity of long-term follow-up remain uncertain and standardized recommendations have not yet been established.

Recently published patient reported outcomes from the SANO trial demonstrated improved short-term health-related quality of life among patients managed with active surveillance compared with immediate surgery, particularly regarding dysphagia, fatigue, dyspnea, and physical functioning [51]. Formal economic analyses suggested a favorable cost-effectiveness of active surveillance compared with immediate surgery [52]. However, intensive surveillance protocols may still impose substantial logistical, psychological, and economic burdens associated with repeated endoscopic and imaging assessments.

Another potential limitation involves the management of locoregional regrowth, which, although often amenable to salvage resection, may present technical challenges and potentially higher postoperative morbidity. Finally, the long-term oncologic safety of surveillance strategies remains under investigation, and current evidence is still largely derived from highly selected patient populations treated in specialized centers.

These considerations highlight that, while active surveillance represents a promising organ-preservation strategy, careful patient selection and rigorous monitoring remain essential to ensure oncologic safety. At present, active surveillance should not be considered the standard of care outside specialized multidisciplinary centers and prospective clinical trials.

## 4. Limitations

Several limitations should be acknowledged for proper interpretation of current evidence regarding active surveillance in esophageal cancer. Most available data derive from highly selected patient populations treated at specialized high-volume centers with strict surveillance protocols, potentially limiting generalizability to routine clinical practice. In addition, long-term oncologic outcomes remain immature, particularly regarding distant recurrence and overall survival. Finally, substantial heterogeneity exists across studies in response-assessment protocols, patient selection, and treatment strategies, limiting direct comparisons between cohorts.

## 5. Conclusions

Non-operative management of esophageal cancer has emerged as a potential organ-preservation strategy supported by evolving clinical evidence. However, the major challenge remains selecting patients who can safely avoid esophagectomy without compromising long-term oncologic outcomes. In addition, the standard follow-up protocol along with the optimal use of molecular biomarkers and evolving systemic therapies remain uncertain. Mature long-term oncologic data are still needed before active surveillance can be broadly adopted in routine clinical practice.

## Figures and Tables

**Table 1:** Summary of major trials in esophageal cancer.

Study	Design	Population	Intervention	Key Findings
**Trials evaluating active surveillance**				
**pre-SANO** [16] (2018)	Prospective cohort	Locally advanced EC after nCRT	Multimodal response evaluation post-nCRT	Established diagnostic strategy to identify clinical complete response. Guides surveillance
**SANO** [18] (2025)	Phase III randomized non-inferiority trial	Patients with cCR after nCRT	Active surveillance vs. immediate surgery post-nCRT	Non- inferior OS (2 year: 74% vs. 71%). Organ preservation in 40–50%
**INFINITY** [22] (2024)	Phase II single-arm	MSI-H GEJ adenocarcinoma	Tremelimumab + durvalumab (neo)	pCR 60%
**Trials with implications for organ preservation**				
**CheckMate 577** [24] (2021)	Phase III RCT	Resectable EC post-nCRT	Adjuvant nivolumab vs. placebo	Significantly improved disease-free survival with nivolumab (22.4 vs. 11 months). Established role of adjuvant immunotherapy
**PALACE-1** [25] (2021)	Phase Ib single-arm	Locally advanced esophageal squamous cell carcinoma	Neoadjuvant pembrolizumab + chemoradiotherapy	pCR ~55%. High R0 resection rates
**ESOPEC** [3] (2024)	Phase III RCT	Resectable EAC	Perioperative FLOT vs. CROSS	Improved overall survival with FLOT (66 vs. 57 months). Suggests perioperative chemotherapy alternative to CROSS in adenocarcinoma
**NEONIPIGA** [26] (2024)	Phase II single-arm	MSI-H GEJ adenocarcinoma	Nivolumab + ipilimumab (neo)	pCR 58.6%
**DANTE** [20] (2024)	Phase II/III	GEJ adenocarcinoma	FLOT vs. FLOT + atezolizumab (peri)	pCR approximately 25% and improved tumor regression grade
**KEYNOTE-585** [19] (2024)	Phase III randomized double-blind controlled trial	Locally advanced resectable gastric or GEJ adenocarcinoma	Perioperative chemotherapy (CF or FLOT) ± pembrolizumab	Increased pCR rates but no significantly improved OS
**MATTERHRON** [21] (2025)	Phase III RCT	GEJ adenocarcinoma	FLOT vs. FLOT + durvalumab (peri)	Improved pCR (19% vs. 7.2%)
**CROSS** [4,5] (2012–2015)	Phase III RCT	Resectable esophageal or GEJ cancer	nCRT + surgery vs. surgery alone	pCR 29% overall (49% in squamous cell carcinoma)

Abbreviations: RCT, randomized controlled trial; EC, esophageal cancer; GEJ, gastroesophageal junction; nCRT, neoadjuvant chemoradiotherapy; MSI-H, microsatellite instability-high; EAC, esophageal adenocarcinoma; ESCC, esophageal squamous cell carcinoma; pCR, pathologic complete response; OS, overall survival; cCR, clinical complete response.

## Data Availability

No new data were created or analyzed in this study. Data sharing is not applicable to this article.
